# Novel Janus Kinase Inhibitors in the Treatment of Dermatologic Conditions

**DOI:** 10.3390/molecules28248064

**Published:** 2023-12-13

**Authors:** Izabella Ryguła, Wojciech Pikiewicz, Konrad Kaminiów

**Affiliations:** 1 Faculty of Medical Sciences in Katowice, Medical University of Silesia, 40-752 Katowice, Poland; izabella.ryg@gmail.com; 2 Department of Medical and Health Sciences, Collegium Medicum—Faculty of Medicine, WSB University, 41-300 Dąbrowa Górnicza, Poland; wojciech.pikiewicz@wsb.edu.pl

**Keywords:** JAK inhibitors, Janus kinase inhibitors, JAKinibs, JAK-STAT pathway, autoimmune skin diseases, inflammatory skin conditions, dermatology

## Abstract

Janus kinase inhibitors, also known as JAK inhibitors, JAKinibs or JAKi, are a new group of disease-modifying drugs. They work by inhibiting enzymes involved in the transmission of information from receptors located in the cell membrane to the cell interior, specifically to the cell nucleus, thus disrupting the JAK-STAT pathway. This pathway plays a role in key cellular processes such as the immune response and cell growth. This feature is used in the treatment of patients with rheumatological, gastroenterological and hematological diseases. Recently, it has been discovered that JAK-STAT pathway inhibitors also show therapeutic potential against dermatological diseases such as atopic dermatitis, psoriasis, alopecia areata and acquired vitiligo. Studies are underway to use them in the treatment of several other dermatoses. Janus kinase inhibitors represent a promising class of drugs for the treatment of skin diseases refractory to conventional therapy. The purpose of this review is to summarize the latest knowledge on the use of JAKi in dermatological treatment.

## 1. Introduction

Tremendous advances in the ability to analyze the immunological patterns and molecular processes leading to specific dermatoses are contributing to the expanding array of therapeutic options available to dermatologists [1,2,3]. Analysis of skin biopsies has made it possible to pinpoint the cytokines, receptors and signaling proteins involved in the development of dermatological conditions [4]. Recent studies show that a huge role in the pathophysiology of cutaneous diseases with an inflammatory/autoimmune basis is played by the cytokine-induced Janus kinase signaling system [5,6,7]. JAKi are a family of non-receptor tyrosine kinases that transmit signals from the cell membrane to the cell nucleus via signal transducers and activators of transcription (STAT) proteins [8]. Awareness of the importance of the JAK/STAT pathway in the pathomechanisms of skin diseases has contributed to the development of a new class of drugs that interfere with this pathway—JAK inhibitors.

## 2. JAK/STAT Pathway

**Figure 1:**
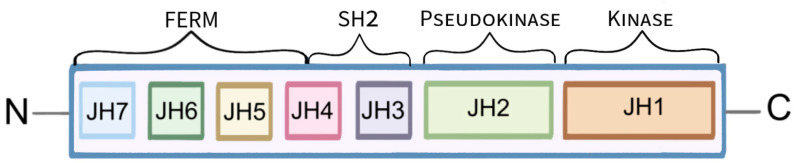
Schematic presentation of the Janus kinase’s structure. The function of the FERM and SH2 domains is to link JAK to receptors. The pseudokinase domain is thought to regulate the activity of the kinase domain, which leads to the phosphorylation of the receptor tyrosine, followed by phosphorylation of downstream molecules.

JAKs are a family of cytoplasmic, non-receptor tyrosine kinases that are composed of seven JAK homology (JH) domains. Functionally, a distinction is made between the FERM domain (JH4, 5, 6 and 7), the Src homology 2 (SH2) domain (JH3 and 4) and the tandem kinase domains: pseudokinase (JH2) and tyrosine kinase (JH1) (Figure 1) [9,10]. The tyrosine kinase domain consists of approximately 250 amino acid residues. JH1 encodes the kinase protein, which is the structure domain of the kinase. It is responsible for substrate phosphorylation, and it is this domain that has become the main target for the introduction of new drug therapies. The pseudokinase domain resembles the kinase domain in its structure but does not exhibit tyrosine kinase activity. The pseudokinase domain is involved in the interaction of JAK and STAT and the inhibition of tyrosine kinase activity by binding to it. The function of the SH2 and FERM domains is to mediate interactions with two intracellular peptide motifs of the cytokine receptor: the proline rich ‘Box1’ and the hydrophobic ‘Box2’ [9]. There are four different Janus kinases: JAK1, JAK2, JAK3 and TYK2 (tyrosine kinase 2) [6,11,12]. Expression of JAK1, JAK2 and TYK2 occurs in many tissues to regulate immunity, while JAK3 is expressed mainly in hematopoietic cells participating in hematopoiesis [13,14,15,16]. The action of JAK is strictly determined by the mediators of inflammation–cytokines: interleukins (IL), interferons (IFN), growth factors along with their receptors with which JAKi are linked [6,14,17,18]. Cytokine-induced signal transport is mediated by different combinations of different types of JAK proteins, for example, the combination of JAK2 and TYK2 is necessary for the action of IL-12 and Il-23 (Table 1) [5,14]. Cytokines bind to the extracellular domains of corresponding receptors located on specific cells leading to conformational changes within the intracellular domain. This results in bringing two JAK molecules close enough to each other that their mutual phosphorylation and activation is feasible [6,11,17]. The activated JAKi then lead to further intracellular signal transduction through phosphorylation and activation of STAT proteins [12]. STAT proteins are signal transducers and activators of transcription that are intracellular transcription factors. The family of these proteins includes seven members: STAT1, STAT2, STAT3, STAT4, STAT5a, STAT5b and STAT6 [13,19]. STATs are involved in many key cellular processes: processes of proliferation, differentiation, apoptosis and functional activation [19,20]. These proteins are composed of an N-terminal domain, a coiled-coil-type domain, a DNA-binding domain, a transcription activation domain, an SH2 domain and a tyrosine activation domain [21]. Activated STAT proteins dimerize and are transported into the cell nucleus to positively or negatively modulate the expression of target genes, encoding, for example, inflammatory cytokines involved in the formation of numerous diseases, including dermatological conditions [13,22].

**Figure 2:**
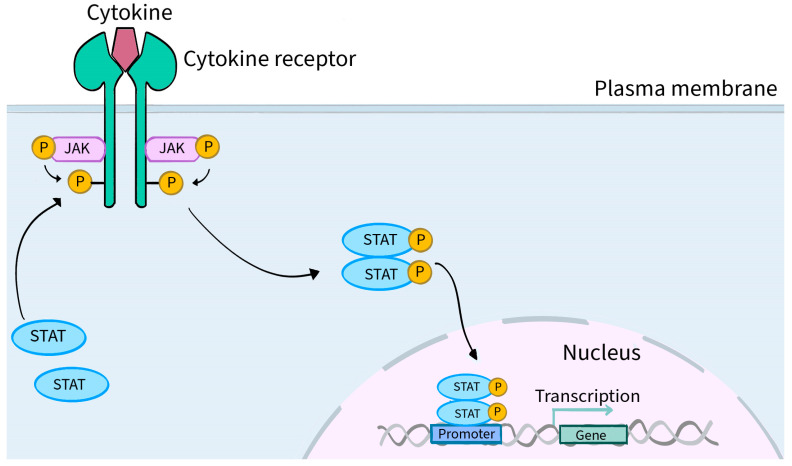
Schematic presentation of JAK-STAT pathway. The attachment of a ligand in the form of a cytokine or hormone (examples: IFN, IL-2, IL-27, IL-19, EPO and OSM) to the extracellular domain of the respective receptors located on specific cells induces conformational changes within their intracellular parts. These changes lead to the two JAK molecules approaching each other, resulting in their phosphorylation (P) and activation. Phosphorylation of the cytoplasmic part of the receptor also occurs, creating a docking site for STAT proteins. STAT proteins, which are signal transducers and activators of transcription, are intracellular transcription factors. STATs bind to the cytoplasmic part of the receptor and their phosphorylation, activation and dimerization occur. A dimer consisting of two STAT molecules translocates into the cell nucleus, where it directly interacts with the DNA matrix and positively or negatively regulates the expression of thousands of different target genes, encoding, for example, inflammatory cytokines that are involved in the pathogenesis of numerous diseases, including dermatological conditions.

In general, the JAK-STAT pathway is a pathway activated by cytokine stimulation that allows signals from outside the cell to pass through the cell membrane to the nucleus, resulting in changes in DNA transcription [14]. Figure 2 shows a schematic presentation of JAK-STAT pathway. The utilization of JAK by various receptors coupled to downstream STAT signal transduction results in a mechanism to achieve exceptional in vivo specificity for more than 60 cytokines and growth factors [11,23].

## 3. Janus Kinase Inhibitors

**Figure 3:**
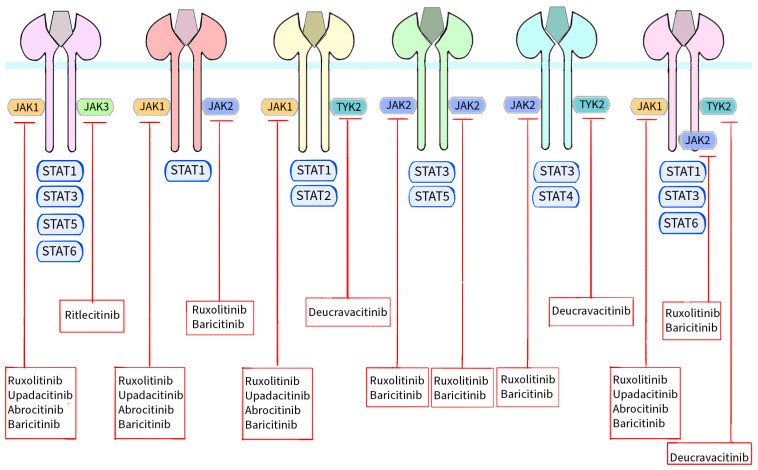
Schematic representation of JAKs with their respective STAT proteins and the site of action of individual JAKs approved by the FDA or EMA for use in the treatment of dermatological conditions. The binding of different ligands to their specific receptor subunits leads to the activation of a specific JAK/STAT pathway. Receptors for cytokines transmit the signal to the cell nucleus via their associated Janus kinases. There are four enzymes in this family: JAK1, JAK2, JAK3 and TYK2. These kinases are essential for signal transduction from cytokine receptors lacking kinase activity. Signal transducers and activators of STAT transcription are also involved in signal transport to the cell nucleus. Seven homologous STAT proteins are currently known: STAT1, STAT2, STAT3, STAT4, STAT5a, STAT5b and STAT6. Each cytokine receptor recruits and activates a specific combination in the JAK/STAT cascades as shown in the figure. Activation results in signal transduction to the cell nucleus, modulation of gene expression and formation of molecules that may be involved in the pathogenesis of skin diseases. However, the signal transduction cascade from the receptor, through JAK/STAT to the cell nucleus, is inhibited by Janus kinase inhibitors. Ruxolitinib and baricitinib are inhibitors of both JAK1 and JAK2, upadacitinib and abrocitinib inhibit JAK1, ritlecitinib blocks JAK3 activity and deucravacitinib inhibits TYK2.

Recognition of the importance of the JAK/STAT pathway in the pathogenesis of many inflammatory and autoimmune diseases has contributed to the development of a new class of drugs—Janus kinase inhibitors. JAKi stop the intracellular signal transduction pathway by inhibiting JAK protein phosphorylation catalyzed by the kinase component of JAK [4]. In September 2021, the Food and Drug Administration (FDA) approved the first JAK inhibitor, ruxolitinib, for the treatment of skin disorders [24]. Since then, more Janus kinase inhibitors have been successively approved for the treatment of dermatoses. The advantage of JAKinibs is that they can be administered by oral or topical routes. This distinguishes them from biologic drugs, which are administered via subcutaneous or intravenous injections. Topical application of JAKi can successfully reduce the risk of side effects compared to their use via the oral route. Noteworthy is the fact that, unlike topical corticosteroids, topical JAKinibs do not cause telangiectasia or skin atrophy [1]. There are two generations of JAKi. Generation I, which includes, for example, ruxolitinib or baricitinib, is characterized by lower specificity toward various Janus kinase isoforms, which is associated with a relatively higher risk of side effects. However, their use can be argued by the theory that blocking multiple JAKi benefits therapeutic success. Second-generation JAKinibs (for example, upadacitinib, abrocitinib, deucravacitinib) are characterized by greater selectivity and specificity. This causes them to be more valued, as their use results in fewer side effects which has an impact on the eventual maintenance of treatment efficacy [4,5,25,26]. Currently, atopic dermatitis, alopecia areata, vitiligo and psoriasis are dermatological conditions for the treatment of which JAKi have been officially approved by the FDA or EMA. In Table 2, we have provided a brief summary of the JAKi and dermatological diseases for which they have been approved by the FDA or EMA [24,27,28]. Figure 3 shows a schematic presentation of Janus kinases together with the STAT proteins with which they interact and the site of action of individual Janus kinase inhibitors [10,12].

## 4. Dermatological Conditions Where JAK Inhibitors Are Approved by the FDA or EMA

### 4.1. Atopic Dermatitis

Atopic dermatitis (AD) is one of the chronic inflammatory dermatoses, with genetic predisposition, abnormal skin barrier function, abnormal microbiome, dysfunctional immune system and environmental factors cited as underlying causes [29]. Chronic, persistent pruritus can significantly reduce a patient’s quality of life or self-esteem, increase the risk of depression or anxiety, and have a negative impact on sleep [30,31]. The diagnosis of AD is relatively more common in the pediatric population, but this skin disease can occur at any age [1]. A key role in the pathogenesis of AD is attributed to a strong activation of the immune response, both in the serum and in the skin, involving Th2 helper lymphocytes with their associated cytokines IL-4, IL-5, IL-13 and IL-31. The cytokines IL-4, IL-13 and IL-31 require further signaling through the JAK/STAT pathway [32]. In Table 3, we discussed the exact importance of these cytokines in the formation of AD [23,33,34,35,36,37,38].

Inhibition of gene expression for filaggrin, involucrin and loricrin via IL-4 and IL-13 promotes skin dehydration and destabilizes skin barrier integrity resulting in dryness and increased likelihood of skin superinfection [38,39]. In addition, modulation of gene expression for cathelicidin and β-defensins (innate immune response genes) potentiates the risk of skin infection by pathogens. This results in exacerbation of AD [36].

It is noteworthy that Th1 lymphocytes are also involved in the pathogenesis of AD along with the cytokine it produces, IFN-γ, and Th17/Th22 lymphocytes along with IL-17 or IL-22. IL-22 plays a role especially in chronic lesions by promoting epidermal hyperplasia [12]. These interleukins also act in a JAK-STAT pathway-dependent manner [33]. Ruxolitinib, upadicitinib, abrocitinib and baricitinib are JAKinibs approved by the FDA or EMA for the treatment of AD.

Ruxolitinib belongs to the first-generation JAKinibs that inhibit JAK1 and JAK2. Two phase 3 trials (this study is registered at ClinicalTrials.gov available online: https://www.clinicaltrials.gov/ (accessed on 2 October 2023)), NCT03745638, NCT03745651) have confirmed the efficacy and safety of ruxolitinib cream in AD in monotherapy. It is recommended to be used continuously for 8 weeks twice daily, and then after continuous treatment, it should be used occasionally as needed for long-term disease control. The low plasma concentration of ruxolitinib suggests that systemic JAK inhibition is highly unlikely in this case. Adverse effects occurred relatively infrequently and were mostly unrelated to treatment [40].

Upadicitinib is a second-generation JAKinib, inhibiting JAK1. Two replicated, randomized, double-blind, controlled phase 3 studies (NCT03569293 and NCT03607422) showed that the use of one upadicitinib tablet per day as a monotherapy is an effective treatment for adolescents and adults with moderate to severe atopic dermatitis in terms of skin symptoms, itching, skin pain and quality of life [41]. In contrast, another phase 3 study (NCT04195698) showed that patients previously treated with dupilumab had more favorable treatment outcomes after changing it to upadicitinib [42]. Upadicitinib has no new side effects compared to other JAK inhibitors, and its safety profile is reasonably acceptable (NCT03569293, NCT03607422, NCT03568318) [43].

Abrocitinib is a second-generation JAK1 inhibitor used for atopic dermatitis (moderate to severe) in the form of 100 mg or 200 mg tablets (one tablet per day). Observations made during the Phase 3 Atopic Dermatitis Efficacy and Safety (JADE) REGIMEN trial (NCT03627767) showed that continuous monotherapy with abrocitinib 200 mg is the therapy with the best results in terms of maintaining disease control. No exacerbation of symptoms occurred in patients treated with the 100 mg dose for the 40 weeks of the trial, so it is believed that induction-maintenance therapy (using abrocitinib 200 mg first and then switching to the 100 mg dose) will be the most rational approach among most patients. On the other hand, in case of possible AD exacerbation during abrocitinib therapy, combination therapy is recommended: abrocitinib 200 mg combined with a topical corticosteroid [44]. Abrocitinib shows superiority over dupilumab, with faster and greater improvement in skin clearance (NCT03720470) [45,46].

Baricitinib is a JAK1 and JAK2 inhibitor. The use of baricitinib in monotherapy at a dose of 4 mg or a reduced dose of 2 mg reduces pruritus, improves skin, sleep and quality of life among patients struggling with moderate to severe atopic dermatitis (NCT03334435) (NCT03334435) [47,48,49]. For baricitinib, the incidence of adverse events of special interest (AESI) is low [50].

In Table 4, we have presented active and completed clinical trials of JAKi for the treatment of atopic dermatitis.

### 4.2. Alopecia Areata

Alopecia areata (AA) is characterized by partial or complete, sudden, non-scarring hair loss with preservation of hair follicles. The incidence ranges from 1.7 to 2.1%, and the first symptoms usually occur before the age of 30 [51,52]. The disease can affect all human hair areas (in both children and adults). AA can be associated with psychological suffering for the patient and a decrease in quality of life, especially when it affects areas such as the scalp, beard, mustache, eyelashes, or eyebrows [53]. Alopecia areata arises from loss of immune privileging in hair follicles during the anagen phase and results in their attack by autoreactive CD8+ T cells and NK T cells [54]. Follicles in AA are characterized by increased expression of MHC class I, MHC class II, elevated levels of IL-2, IL-15 and CXCL belonging to the pro-inflammatory interleukin family, and abundant infiltration of various inflammatory cells [55,56]. CD8+ T lymphocytes, upon activation by NK cells via the NKG2D receptor, produce IFNγ mediated by JAK1 and JAK3. Interferon stimulates IL-15 secretion via follicular epithelial cells using JAK1 and JAK2 signaling. Interleukin-15 affects CD8+ T lymphocytes, also through the JAK-STAT pathway, resulting in the secretion of perforin and granzymes by these lymphocytes. The result of these processes is hair follicle dystrophy and premature onset of the catagen phase resulting in alopecia [57,58,59].

Janus kinase inhibitors are a kind of breakthrough in the treatment of alopecia areata. Baricitinib and ritlecitinib are the first and, so far, only drugs approved by the FDA for the treatment of AA. Baricitinib has found use for treating the disease among adult patients (≥18 yo), while ritlecitinib can be used in both adult and adolescent patients (≥12 yo). It is noteworthy that the research on these two formulations was conducted by a single doctor—Dr. Brett King from Yale School of Medicine [60].

Baricitinib is a first-generation JAKinib that inhibits JAK1 and JAK2 [4]. Two randomized, placebo-controlled phase 3 trials conducted by a team led by Dr. Brett King showed that oral baricitinib administered once daily had hair regrowth efficacy compared to the control group after 36 weeks of use. The percentage of patients with a SALT score ≤20 at 36 weeks of use in the BRAVE-AA1 trial (NCT03570749) was 38.8% for the 4 mg dose of baricitinib, 22.8% for the 2 mg drug and 6.2% for placebo, and for the BRAVE-AA2 trial (NCT03899259) the percentages were 35.9%, 19.4% and 3.3%, respectively. Acne, increased cholesterol and creatine kinase levels were relatively more common with baricitinib than placebo [61].

Ritlecitinib belongs to the second-generation inhibitors that irreversibly inhibit JAK3 [62]. A formulation containing this active ingredient was relatively recently approved for the treatment of AA: the FDA approved it in June 2023 and the EMA in September 2023. A phase 3 trial lasting 48 weeks, also supervised by Dr. King, showed ritlecitinib to be effective in treating AA and well tolerated among the population aged 12 years and older. Doses of 30 mg and 50 mg taken once daily (with or without a saturating dose of 200 mg taken over four weeks) resulted in significant hair regrowth compared with the control group. The drug was generally safe, and major adverse cardiovascular events, opportunistic infections or deaths were reported throughout the study period (NCT03732807) [63]. A long-term evaluation of ritlecitinib is underway: NCT04006457.

In Table 5 we have presented active and completed clinical trials of JAKi for the treatment of alopecia areata.

### 4.3. Non-Segmental Vitiligo

Acquired vitiligo involves the formation of well-demarcated, discolored patches on the skin of any part of the body as a result of the loss of melanocytes within the epidermis. This dermatosis affects about 1–2% of the human population. Non-segmental vitiligo clinically occupies the skin surface regardless of the dermatomes. Skin lesions in the course of vitiligo impinge on the patient’s quality of life, leading to psychic discomfort, social withdrawal and stigmatization [64,65,66,67,68]. Certain exogenous and/or endogenous factors in genetically predisposed individuals lead to cellular stress within melanocytes, which promotes the migration of CD8+ T lymphocytes into the epidermis. CD8+ T lymphocytes are responsible for perforin- and granzyme-mediated destruction of melanocytes. These lymphocytes are also responsible for the local production of disease-promoting proteins: interferon gamma and tumor necrosis factor alpha. IFN-γ causes activation of the JAK/STAT pathway in nearby keratinocytes leading to increased levels of the chemokines CXCL9 and CXCL10. It is worth noting that CXCL10 binds to the CXCR3 receptor located on CD8+ T cells—an example of positive feedback. The CXCL10/CXCR3 axis is involved in recruiting more T cells to the skin, exacerbating inflammation. Interferon-gamma is responsible for inhibiting melanogenesis and inducing melanocyte apoptosis. IFN-γ, along with its associated heterodimer: JAK1-JAK2, plays an important role in the pathogenesis of vitiligo [69,70,71,72,73].

Ruxolitinib is the first and only FDA-approved pharmacological drug for the treatment of non-segmental vitiligo. It belongs to the first generation JAK1 and JAK2 inhibitors. Two randomized phase 3 trials (NCT04052425 and NCT04057573) were conducted in which patients in the study group were applied 1.5% ruxolitinib cream twice daily for 52 weeks. This ultimately resulted in relatively greater repigmentation of lesions compared to the control group. However, it is noteworthy that patients developed acne and pruritus at the application site [74,75].

In Table 6 we have presented active and completed clinical trials of JAKi for the treatment of vitiligo.

### 4.4. Psoriasis

Psoriasis (PsO) is an inflammatory erythematous and scaly skin disease that affects about 2% of the population. It has been recognized by the World Health Organization as a serious non-communicable disease, and the continued increase in its incidence is a public health concern. The course of ordinary (plaque-like) PsO results in characteristic sharply demarcated erythematous, itchy and scaly lesions [23,76,77,78]. PsO is characterized by the properties of an autoimmune disease on (auto)inflammatory grounds [79] Activated myeloid dendritic cells secrete TNF-α, IL-23 and IL-12, the latter two interleukins affecting Th17 and Th1 proliferation. This results in an accumulation of Th17 and Th1 lymphocytes within the lesions and their secretion of IL-17, IL-21 and IL-22 (Th17) and IFNγ (Th1). It is worth noting that IL-23, for example, promotes Th17 proliferation precisely through JAK1/JAK2/TYK2 signaling. Finally, IL-22, after binding to the surface receptors IL-10R2 and IL-22R1, leads to acanthosis of keratinocytes also through the JAK/STAT pathway, more specifically with the participation of JAK1/TYK2 and STAT3. In addition, IL-21 and IL-6, which are present around psoriatic lesions, stimulate Th-17 to produce IL-17 through a JAK-STAT signaling-dependent pathway [80,81,82,83,84,85].

Deucravacitinib is a TYK2 inhibitor approved by the FDA and EMA for the treatment of PsO. In the randomized phase 3 PETYK PSO-1 trial (NCT03624127), participants were assigned to a group receiving deucravacitinib 6 mg once daily, to a group receiving apremilast 30 mg daily, or to a placebo group. At week 16, the response rate for PASI 75 was relatively higher for the deucravacitinib-treated group than for the apremilast-treated group and the placebo group, 58.4%, 35.1% and 12.7%, respectively. Efficacy was maintained until the 52nd week of the study. The most common side effects among patients using deucravactinib were nasopharyngitis (6.3%) and upper respiratory tract infection (6.3%) [86].

In Table 7 we have presented active and completed clinical trials of JAKi for the treatment of psoriasis.

### 4.5. JAK Inhibitors in Other Dermatology Conditions

The JAK/STAT pathway is involved in the pathogenesis of many other diseases manifested by skin lesions. Studies are underway to test the therapeutic potential of Janus kinase inhibitors in such dermatological conditions as: hidradenitis suppurativa, chronic hand eczema, diffuse cutaneous systemic scleroderma, granuloma annulare, dermatomyositis, lichen planus and lupus erythematosus. In Table 8, we preface current and completed clinical trials on the therapeutic value of JAKi in these conditions.

## 5. Side Effects of Janus Kinase Inhibitors

What researchers always pay attention to, in addition to the effectiveness of a method, are its side effects. In 2019, the FDA added boxed warnings (formerly known as Black Box Warnings) regarding the increased risk of blood clots and death during oral use of tofacitinib 10 mg twice daily in patients with ulcerative colitis [87]. In the ORAL Surveillance study (NCT02092467), the incidence of cancer and major adverse cardiovascular events (MACE) were compared among groups of patients receiving tofacitinib 5 mg twice daily, tofacitinib 10 mg twice daily and a tumor necrosis factor inhibitor. All patients had active rheumatoid arthritis, had at least one additional cardiovascular risk factor and were aged 50 years or older. The final results showed that the risk of MACE and cancer was relatively higher for the combined doses of tofacitinib (3.4% and 4.2%, respectively) than for the TNF inhibitor (2.5% and 2.9%) [88]. Post hoc analysis of this study showed that the presence at baseline of risk factors such as smoking, age > 65 years, taking oral contraceptives/hormone replacement therapy and venous thromboembolism (VTE), coronary artery disease or a history of hypertension resulted in an increased risk of VTE or MACE among patients taking JAKi therapy [89,90]. This information raises questions about the advantage of benefits over risks in treating dermatological conditions with Janus kinase inhibitors. However, it is noteworthy that the population of patients with dermatological conditions is relatively younger compared to those suffering from rheumatoid arthritis. It is also worth noting that a large cohort study that included 158,123 patients showed that chronic inflammatory skin diseases, including psoriasis, alopecia areata, vitiligo and atopic dermatitis were not associated with an increased incidence of VTE after controlling for relevant VTE risk factors [91]. In Table 9, we collected patient-reported selected adverse reactions in clinical phase 3 trials that had been ongoing on FDA- or EMA-approved JAKs for the treatment of dermatological conditions: ruxolitinib, upadacitinib, abrocitinib, baricitinib, deucravacitinib and ritlecitinib [27,40,41,44,47,48,61,63,74,75,86,92,93,94,95,96]. It is very essential that dermatologists thoroughly conduct a subject and physical examination of the patient. This will allow them to assess the patient’s comorbidities, current condition and the medications he is taking. This knowledge will make it possible to estimate as accurately as possible whether the introduction of JAKi into therapy in a given case will bring more benefits or risks [27]. However, of great note is the fact that severe adverse events during the use of JAKi in dermatoses are rare, and common side effects, which include nasopharyngitis, nausea, headache and others are easily manageable and should not pose a risk to the patient.

## 6. Conclusions

The JAK-STAT pathway plays a huge role in the pathogenesis of many conditions, including dermatological diseases. Awareness of the importance of this pathway has led to the development in recent years of a new class of drugs—Janus kinase inhibitors. Undoubtedly, JAK Inhibitors expand the range of available therapeutic options for many dermatological conditions. It is important to remember that dermatological diseases are not only an aesthetic problem, but mainly, and perhaps primarily, conditions that reduce quality of life, satisfaction with one’s appearance and sense of self-confidence. This can be associated with impaired social functioning and depressed mood in these patients, so effective treatment appears to be crucial to maintaining the physical and psychological well-being of patients. JAKinibs represent a promising class of drugs due to the fact that they tend to act quickly, their route of administration is not injection, they have a relatively favorable safety profile and, most importantly, they serve as an effective alternative for patients among whom other therapies have failed. As we have shown above, numerous studies are currently underway to expand the indications for the use of currently approved JAKi, as well as to introduce new Janus kinase inhibitors, creating new opportunities to provide therapy in atopic dermatitis, psoriasis, alopecia areata and non-segmental vitiligo. Moreover, numerous studies on the effectiveness of this group of drugs in hidradenitis suppurativa, dermatomyositis and others are enthusiastically underway. This gives hope to patients for effective treatment of their form of the disease. The increase in the prevalence of the dermatological conditions we have described in this article, as well as other diseases, will drive scientific efforts on the efficacy, use and safety of JAK inhibitors in the coming years. They will undoubtedly find a place in the treatment process, either used in high doses during active treatment, in lower doses as chronic treatment or in combination with other drugs. Of course, it should be kept in mind that, as in all of medicine, drugs used to treat dermatological conditions, in our case, Janus kinase inhibitors, have their side effects, so it seems important to scientifically determine the dosage and safety profile to achieve an optimal therapeutic effect. However, it needs to be added that due to the fact that JAKi are relatively new drugs, a huge role is played by healthcare providers, who should carefully analyze each patient’s risk factors before introducing such therapy and follow strict guidelines. Ongoing research on JAKinibs will allow further development of this branch of pharmacotherapy. We believe that despite the fact that some time has passed since the FDA approved the first JAKi (ruxolitinib—November 2011), as well as the first approval of JAKi for the treatment of dermatological conditions (ruxolitinib for the treatment of atopic dermatitis; September 2021), JAKi are still drugs whose development needs to be watched closely, as they may prove to be major players in the market among dermatological patients.

## Figures and Tables

**Table 1:** Cytokines and hormones that transmit signals via appropriate combinations of Janus kinases.

Kinases	Cytokines or Hormones
JAK1, JAK3	IL-2, IL-4, IL-7, IL-9, IL-15, IL-21, TSLP
JAK1, JAK2	IFNγ, IL-27, IL-31, IL-35
JAK1, TYK2	IFNα, IFNβ, IFNκ, IFNω, IFNε, IFNλ, IL-10, IL-19, IL-20, IL-22, IL-24, IL-26
JAK2, JAK2	EPO, TPO, G-CSF, GM-CSF, GH, Leptin, IL-3, IL-5
JAK2, TYK2	IL-12, IL-23
JAK1, JAK2, TYK2	OSM, LIF, IL-6, IL-11, IL-13

**Abbreviations:** JAK = Janus-activated kinases; TYK = tyrosine kinase; IL = interleukin; TSLP = thymic stromal lymphopoietin; IFN = interferon; EPO = erythropoietin; TPO = thrombopoietin; G-CSF = granulocyte colony-stimulating factor; GM-CSF = granulocyte-macrophage colony-stimulating factor; GH = growth hormone; OSM = oncostatin M; LIF = leukemia inhibitory factor.

**Table 2:** JAK inhibitors and dermatological conditions in which JAKi are approved by the FDA or EMA.

JAK Inhibitors	Generation	Target	Form	Route of Administration	FDA Approved Dermatological Condition	EMA Approved Dermatological Condition
Ruxolitinib	1st	JAK1, JAK2	Cream 1.5%	Topical	Atopic dermatitis (mild to moderate) Vitiligo (non-segmental)	-
Upadicitinib	2nd	JAK1	Tablets 15 mg and 30 mg	Oral	Atopic dermatitis (moderate to severe)	Atopic dermatitis (moderate to severe)
Abrocitinib	2nd	JAK1	Tablets 100 mg and 200 mg	Oral	Atopic dermatitis (moderate to severe)	Atopic dermatitis (moderate to severe)
Baricitinib	1st	JAK1, JAK2	Tablets 2 mg and 4 mg	Oral	Alopecia areata	Alopecia areata Atopic dermatitis (moderate to severe)
Deucravacitinib	2nd	TYK2	Tablets 6 mg	Oral	Psoriasis	Psoriasis
Ritlecitinib	2nd	JAK3	Tablets 50 mg	Oral	Alopecia areata	Alopecia areata

**Abbreviations:** JAK = Janus-activated kinases; TYK = Tyrosine kinase; FDA = Food and Drug Administration; EMA = European Medicines Agency.

**Table 3:** Functions of Th2-related cytokines in the pathogenesis of atopic dermatitis.

Cytokine	Importance in Atopic Dermatitis	Janus Kinase That Transmits Signal to the Cell Nucleus
IL-4	Inhibition of gene expression for filaggrin, loricrin, involucrin and lipid components of the skin barrier Pruritus Modulation of gene expression of cathelicidin and β-defensins	JAK1, JAK3
IL-5	Eosinophilia activator	-
IL-13	Inhibition of gene expression for filaggrin, loricrin, involucrin and lipid components of the skin barrier Pruritus Modulation of gene expression of cathelicidin and β-defensins	JAK1, JAK2, TYK2
IL-31	Pruritus	JAK1, JAK2

**Abbreviations:** JAK = Janus-activated kinases; TYK = tyrosine kinase; IL = interleukin.

**Table 4:** Active and completed clinical trials conducted on JAKi for the treatment of atopic dermatitis according to ClinicalTrials.gov.

Janus Inhibitor	Age of Group	Target	Administration	Phase	Study Number	Sponsor
**Active Clinical Trials**						
Ruxolitinib	≥12 yo–<18 yo	JAK1, JAK2	Topical	Phase 3	NCT05456529	Incyte Corporation
Ruxolitinib	2 yo–11 yo	JAK1, JAK2	Topical	Phase 3	NCT04921969	Incyte Corporation
Upadacitinib	2 yo–12 yo	JAK1	Oral	Phase 1	NCT03646604	AbbVie
Upadacitinib	12 yo–64 yo	JAK1	Oral	Phase 3	NCT05601882	AbbVie
Upadacitinib	12 yo–75 yo	JAK1	Oral	Phase 3	NCT03569293	AbbVie
Upadacitinib	12 yo–75 yo	JAK1	Oral	Phase 3	NCT03607422	AbbVie
Upadacitinib	12 yo–75 yo	JAK1	Oral	Phase 3	NCT03568318	AbbVie
Upadacitinib	18 yo–64 yo	JAK1	Oral	Phase 4	NCT05507580	AbbVie
Upadacitinib	≥18 yo	JAK1	Oral	-	NCT05989932	SIDeMaST
Abrocitinib	≥12 yo	JAK1	Oral	Phase 3	NCT03422822	Pfizer
Abrocitinib	≥18 yo	JAK1	Oral	-	NCT05250115	Pfizer
Abrocitinib	≥12 yo	JAK1	Oral	-	NCT05391061	Pfizer
Abrocitinib	≥0 yo	JAK1	Oral	-	NCT05721937	Pfizer
Abrocitinib	≥18 yo	JAK1	Oral	-	NCT05689151	Pfizer
Abrocitinib	≥18 yo	JAK1	Oral	Phase 4	NCT05602207	Innovaderm Research Inc.
Tofacitinib	≥18 yo	JAK1, JAK3	Topical	Phase 2	NCT05487963	CAGE Bio Inc.
Tofacitinib	12 yo–50 yo (patients with Down Syndrome)	JAK1, JAK3	Oral	Phase 2	NCT04246372	University of Colorado, Denver
Baricitinib	2 yo–17 yo	JAK1, JAK2	Oral	Phase 3	NCT03952559	Eli Lilly and Company
Baricitinib	18 yo–75 yo	JAK1, JAK2	Oral	-	NCT05969730	Mazandaran University of Medical Sciences
**Completed clinical trials**						
Ruxolitinib	≥2 yo–17 yo	JAK1, JAK2	Topical	Phase 1	NCT03257644	Incyte Corporation
Ruxolitinib	12 yo–65 yo	JAK1, JAK2	Topical	Phase 1	NCT03920852	Incyte Corporation
Ruxolitinib	2 yo–11 yo	JAK1, JAK2	Topical	Phase 1	NCT05034822	Incyte Corporation
Ruxolitinib	18 yo–70 yo	JAK1, JAK2	Topical	Phase 2	NCT03011892	Incyte Corporation
Ruxolitinib	18 yo–65 yo	JAK1, JAK2	Topical	Phase 2	NCT04839380	Incyte Corporation
Ruxolitinib	≥12 yo	JAK1, JAK2	Topical	Phase 3	NCT03745638	Incyte Corporation
Ruxolitinib	≥12 yo–17 yo	JAK1, JAK2	Topical	Phase 3	NCT03745651	Incyte Corporation
Tofacitinib	18 yo–60 yo	JAK1, JAK3	Oral	Phase 2	NCT02001181	Pfizer
Upadacitinib	18 yo–75 yo	JAK1	Oral	Phase 2	NCT02925117	AbbVie
Upadacitinib	12 yo–75 yo	JAK1	Oral	Phase 2	NCT03661138	AbbVie
Upadacitinib	18 yo–75 yo	JAK1	Oral	Phase 3	NCT04195698	AbbVie
Upadacitinib	18 yo–75 yo	JAK1	Oral	Phase 3	NCT03738397	AbbVie
Abrocitinib	≥18 yo	JAK1	Oral	Phase 3	NCT04345367	Pfizer
Baricitinib	≥18 yo	JAK1, JAK2	Oral	Phase 2	NCT02576938	Eli Lilly and Company
Baricitinib	≥18 yo	JAK1, JAK2	Oral	Phase 3	NCT03334422	Eli Lilly and Company
Baricitinib	≥18 yo	JAK1, JAK2	Oral	Phase 3	NCT03435081	Eli Lilly and Company
Baricitinib	≥18 yo	JAK1, JAK2	Oral	Phase 3	NCT03334396	Eli Lilly and Company
Baricitinib	≥18 yo	JAK1, JAK2	Oral	Phase 3	NCT03733301	Eli Lilly and Company
Baricitinib	≥18 yo	JAK1, JAK2	Oral	Phase 3	NCT03428100	Eli Lilly and Company
Delgocitinib	≥2 yo	JAK1, JAK2, JAK3, TYK2	Topical	Phase 1	NCT03826901	LEO Pharma
Delgocitinib	≥18 yo	JAK1, JAK2, JAK3, TYK2	Topical	Phase 2	NCT03725722	LEO Pharma
Jaktinib	18 yo–65 yo	JAK1, JAK2, JAK3, TYK2	Oral	Phase 2	NCT04539639	Suzhou Zelgen Biopharmaceuticals Co., Ltd.

**Abbreviations:** JAK = Janus-activated kinases; TYK = tyrosine kinase; yo = years old.

**Table 5:** Active and completed clinical trials conducted on JAKi for the treatment of alopecia areata according to ClinicalTrials.gov.

Janus Inhibitor	Age of Group	Target	Administration	Phase	Study Number	Sponsor
**Active Clinical Trials**						
PF-06651600	≥12 yo	JAK3	Oral	Phase 3	NCT04006457	Pfizer
Baricitinib	18 yo–70 yo	JAK1, JAK2	Oral	Phase 3	NCT03899259	Eli Lilly and Company
Baricitinib	18 yo–70	JAK1, JAK2	Oral	Phase 2/3	NCT03570749	Eli Lilly and Company
Jaktinib	18 yo–65 yo	JAK1, JAK2, JAK3	Topical	Phase 1/2	NCT04445363	Suzhou Zelgen Biopharmaceuticals Co., Ltd.
Jaktinib	18 yo–65 yo	JAK1, JAK2, JAK3	Oral	Phase 3	NCT05255237	Suzhou Zelgen Biopharmaceuticals Co., Ltd.
Tofacitinib	12 yo–50 yo (patients with Down Syndrome)	JAK1, JAK3	Oral	Phase 2	NCT04246372	University of Colorado, Denver
Upadacitinib	12 yo–63 yo	JAK1	Oral	Phase 3	NCT06012240	AbbVie
**Completed clinical trials**						
Delgocitinib	≥18 yo	JAK1, JAK2, JAK3, TYK2	Topical	Phase 2	NCT05332366	LEO Pharma
Jaktinib	≥12 yo	JAK1, JAK2, JAK3	Oral	Phase 2	NCT04034134	Suzhou Zelgen Biopharmaceuticals Co., Ltd.
Ruxolitinib	18 yo–75 yo	JAK1, JAK2	Oral	Phase 2	NCT01950780	Columbia University
Tofacitinib	18 yo–65 yo	JAK1, JAK3	Oral	Phase 2	NCT02299297	Columbia University
Tofacitinib	≥18 yo	JAK1, JAK3	Oral	Phase 2	NCT02812342	Yale University
Tofacitinib	18 yo–90 yo	JAK1, JAK3	Oral	Phase 2	NCT02197455	Yale University
Tofacitinib	18 yo–60 yo	JAK1, JAK3	Oral	Phase 4	NCT03800979	Institute of Dermatology, Thailand
Tofacitinib	≥18 yo	JAK1, JAK3	Oral	-	NCT02312882	Stanford University
PF-06700841	≥18 yo	JAK1, TYK2	Oral	Phase 2	NCT05076006	Emma Guttman

**Abbreviations**: JAK = Janus-activated kinases; TYK = tyrosine kinase; yo = years old.

**Table 6:** Active and completed clinical trials conducted on JAKi for the treatment of vitiligo according to ClinicalTrials.gov.

Janus Inhibitor	Age of Group	Target	Administration	Phase	Study Number	Sponsor
**Active Clinical Trials**						
Baricitinib	≥12 yo	JAK1, JAK2	Oral	-	NCT05950542	Assiut University
Ritlecitinib	≥18 yo	JAK3	Oral	Phase 3	NCT06072183	Pfizer
Ritlecitinib	≥12 yo	JAK3	Oral	Phase 3	NCT05583526	Pfizer
Ruxolitinib	12 yo–99 yo	JAK1, JAK2	Topical	Phase 2	NCT05247489	Incyte Corporation
Ruxolitinib	≥18 yo	JAK1, JAK2	Topical	Phase 2	NCT05750823	Incyte Corporation
Tofacitinib	12 yo–50 yo (patients with Down Syndrome)	JAK1, JAK3	Oral	Phase 2	NCT04246372	University of Colorado, Denver
**Completed clinical trials**						
Baricitinib	18 yo–75 yo	JAK1, JAK2	Oral	Phase 2	NCT04822584	University Hospital, Bordeaux
Ruxolitinib	≥18 yo	JAK1, JAK2	Topical	Phase 2	NCT04896385	Incyte Corporation
Ruxolitinib	18 yo–75 yo	JAK1, JAK2	Topical	Phase 2	NCT03099304	Incyte Corporation
Ruxolitinib	≥12 yo	JAK1, JAK2	Topical	Phase 3	NCT04057573	Incyte Corporation
Ruxolitinib	≥12 yo	JAK1, JAK2	Topical	Phase 3	NCT04530344	Incyte Corporation
Ruxolitinib	≥12 yo	JAK1, JAK2	Topical	Phase 3	NCT04052425	Incyte Corporation
Upadacitinib	18 yo–65 yo	JAK1	Oral	Phase 2	NCT04927975	AbbVie

**Abbreviations**: JAK = Janus-activated kinases; TYK = tyrosine kinase; yo = years old.

**Table 7:** Active and selected completed research on JAKi for the treatment of psoriasis according to ClinicalTrials.gov.

Janus Inhibitor	Age of Group	Target	Administration	Phase	Study Number	Sponsor
**Active Clinical Trials**						
Deucravacitinib	≥18 yo	TYK2	Oral	Phase 4	NCT05478499	Bristol-Myers Squibb
Deucravacitinib	18 yo–75 yo	TYK2	Oral	Phase 4	NCT05858645	University of California, San Francisco
Deucravacitinib	≥18 yo	TYK2	Oral	-	NCT06104644	Bristol-Myers Squibb
Jaktinib	18 yo–65 yo	JAK1, JAK2, JAK3	Oral	Phase 2	NCT04612699	Suzhou Zelgen Biopharmaceuticals Co., Ltd.
Tofacitinib	12 yo–50 yo (patients with Down Syndrome)	JAK1, JAK3	Oral	Phase 2	NCT04246372	University of Colorado, Denver
**Completed clinical trials**						
Baricitinib	≥18 yo	JAK1, JAK2	Oral	Phase 2	NCT01490632	Eli Lilly and Company
Ruxolitinib	18 yo–65 yo	JAK1, JAK2	Oral	Phase 2	NCT00617994	Incyte Corporation
Ruxolitinib	18 yo–75 yo	JAK1, JAK2	Topical	Phase 2	NCT00820950	Incyte Corporation
Ruxolitinib	18 yo–75 yo	JAK1, JAK2	Topical	Phase 2	NCT00778700	Incyte Corporation
Tofacitinib	18 yo–65 yo	JAK1, JAK3	Oral	Phase 1	NCT01736696	Pfizer
Tofacitinib	≥18 yo	JAK1, JAK3	Topical	Phase 2	NCT01831466	Pfizer
Tofacitinib	≥18 yo	JAK1, JAK3	Oral	Phase 2	NCT01710046	Pfizer
Tofacitinib	≥18 yo	JAK1, JAK3	Oral	Phase 3	NCT01882439	Pfizer
PF-06826647	18 yo–55 yo	TYK2	Oral	Phase 1	NCT03210961	Pfizer
PF-06263276	≥18 yo	JAK1, JAK2, JAK3, TYK2	Topical	Phase 1	NCT02193815	Pfizer
PF-06700841	18 yo–75 yo	JAK1, TYK2	Oral	Phase 2	NCT02969018	Pfizer
CP-690,550	18 yo–65 yo	JAK1, JAK2, JAK3	Oral	Phase 2	NCT00678561	Pfizer
CP-690-550	18 yo–99 yo	JAK1, JAK2, JAK3	Oral	Phase 2	NCT01246583	Pfizer
CP-690,550	≥18 yo	JAK1, JAK2, JAK3	Oral	Phase 3	NCT01815424	Pfizer
CP-690,550	≥18 yo	JAK1, JAK2, JAK3	Oral	Phase 3	NCT01309737	Pfizer
CP-690,550	≥18 yo	JAK1, JAK2, JAK3	Oral	Phase 3	NCT01276639	Pfizer
CP-690,550	≥18 yo	JAK1, JAK2, JAK3	Oral	Phase 3	NCT01186744	Pfizer
CP-690,550	≥20 yo	JAK1, JAK2, JAK3	Oral	Phase 3	NCT01519089	Pfizer

**Abbreviations:** JAK = Janus-activated kinases; TYK = tyrosine kinase; yo = years old.

**Table 8:** Active and completed clinical trials conducted on the use of JAK inhibitors w hidradenitis suppurativa, chronic hand eczema, diffuse cutaneous systemic scleroderma, granuloma annulare, dermatomyositis, lichen planus and lupus erythematosus according to ClinicalTrials.gov.

Dermatological Condition	JAK Inhibitor	Target	Administration	Status	Phase	Study Number	Sponsor
Hidradenitis suppurativa	Tofacitinib	JAK1, JAK3	Oral	Active	Phase 2	NCT04246372	University of Colorado, Denver
	Upadacitinib	JAK1	Oral	Active	Phase 3	NCT05889182	AbbVie
	Deucravacitinib	TYK2	Oral	Active	Phase 2	NCT05997277	Beth Israel Deaconess Medical Center
	Upadacitinib	JAK1	Oral	Completed	Phase 2	NCT04430855	AbbVie
	INCB054707	JAK1	Oral	Completed	Phase 2	NCT03607487	Incyte Corporation
Chronic hand eczema	Ruxolitinib	JAK1, JAK2	Topical	Active	Phase 2	NCT05906628	Incyte Corporation
	Delgocitinib	JAK1, JAK2, JAK3, TYK2	Topical	Completed	Phase 2	NCT03683719	LEO Pharma
Diffuse cutaneous systemic scleroderma	Tofacitinib	JAK1, JAK3	Oral	Active	Phase 2	NCT06044844	Bangabandhu Sheikh Mujib Medical University, Dhaka, Bangladesh
	Tofacitinib	JAK1, JAK3	Oral	Completed	Phase 1/2	NCT03274076	University of Michigan
Granuloma Annulare	AC-1101	JAK1, JAK3	Topical	Active	Phase 1	NCT05580042	TWi Biotechnology, Inc.
	Abrocitinib	JAK1	Oral	Active	Phase 2	NCT05650736	William Damsky
	Tofacitinib	JAK1, JAK3	Oral	Completed	Phase 1	NCT03910543	Yale University
Dermatomyositis	Tofacitinib	JAK1, JAK3	Oral	Completed	Phase 1	NCT03002649	Johns Hopkins University
	Baricitinib	JAK1, JAK3	Oral	Active	Phase 3	NCT04972760	Assistance Publique—Hôpitaux de Paris
	Baricitinib	JAK1, JAK3	Oral	Active	Phase 2	NCT05524311	Assistance Publique—Hôpitaux de Paris
	Brepocitinib	JAK1, TYK2	Oral	Active	Phase 3	NCT05437263	Priovant Therapeutics, Inc.
	Baricitinib	JAK1, JAK2	Oral	Completed	Phase 2	NCT05188521	Aaron R. Mangold
Lupus erythematosus	Deucravacitinib	TYK2	Topical	Active	Phase 3	NCT05620407	Bristol-Myers Squibb
	Deucravactinib	TYK2	Topical	Active	Phase 3	NCT05617677	Bristol-Myers Squibb
	Upadacitinib	JAK1	Oral	Active	Phase 3	NCT05843643	AbbVie
	Tofacitinib	JAK3, JAK1	Oral	Active	Phase 1	NCT05048238	National Institute of Allergy and Infectious Diseases (NIAID)
	Tofacitinib	JAK3, JAK1	Oral	Completed	Phase 1	NCT02535689	National Institute of Arthritis and Musculoskeletal and Skin Diseases (NIAMS)
	Tofacitinib	JAK3, JAK1	Oral	Completed	Phase 2	NCT03288324	Children’s Hospital Medical Center, Cincinnati
	Delgocitinib	JAK1, JAK2, JAK3, TYK2	Topical	Completed	Phase 2	NCT03958955	LEO Pharma
	Baricitinib	JAK1, JAK2	Oral	Completed	Phase 2	NCT02708095	Eli Lilly and Company
	Baricitinib	JAK1, JAK2	Oral	Completed	Phase 3	NCT03616912	Eli Lilly and Company
	Baricitinib	JAK1, JAK2	Oral	Completed	Phase 3	NCT03843125	Eli Lilly and Company
	Baricitinib	JAK1, JAK2	Oral	Completed	Phase 3	NCT03616964	Eli Lilly and Company

**Abbreviations:** JAK = Janus-activated kinases; TYK = tyrosine kinase.

**Table 9:** Selected side effects after the treatment of dermatological conditions using Janus kinase inhibitors approved by the FDA or EMA.

**Selected Side Effects after the Treatment of Dermatological Conditions of Oral Janus Kinase Inhibitors**	
Infections	Upper respiratory infections
	Nasopharyngitis
	Herpes Simplex reactivation
	Herpes Zoster reactivation
	Urinary tract infections
	Serious infection
Gastrointestinal disorders	Nausea
	Diarrhea
Neurological disorders	Headache
	Dizziness
Skin side effects	Acne
	Itching
	Folliculitis
Laboratory abnormalities	Elevated creatine phosphokinase levels
	Increased levels of cholesterol and low- and high-density lipoproteins
	Neutropenia
	Thrombocytosis
Venous thromboembolism	
Tumors	
**Selected side effects after the treatment of dermatological conditions of topical Janus kinase inhibitors**	
Neutropenia	
Oral herpes	
Application site pain	
Application site pruritus	
Skin bacterial infection	
Alopecia	
Application site erythema	
Skin papilloma	

## Data Availability

Not applicable.
